# Prognostic implication and functional annotations of APOBEC3G expression in patients with Melanoma

**DOI:** 10.7150/jca.46383

**Published:** 2020-07-06

**Authors:** Wei Han, Jun Xu, Guo-Liang Shen

**Affiliations:** 1 Department of Burn and Plastic Surgery , The First Affiliated Hospital of Soochow University, Suzhou, P.R. China, 215000.; 2 Department of Surgery, Soochow University, Suzhou, P.R. China, 215000.

**Keywords:** APOBEC3G, melanoma, immune, prognosis

## Abstract

**Aim:** Skin cutaneous melanoma (SKCM) is one of the most life-threatening malignancies damaging human health. APOBEC3G (A3G) has been found in several cancers; however, the role of A3G in SKCM is rarely studied. This study aimed to investigate the expression of A3G in tumor tissue and its prognostic value in SKCM patients.

**Method:** A total of 512 SKCM patients from the First Affiliated Hospital of Soochow University (FAHSU) and the Cancer Genome Atlas (TCGA) database were consecutively recruited in analyses. Differential transcriptional and proteome expression profiles were obtained from multiple datasets. GEPIA was used to assess the survival analysis between distinguished groups. Both univariate and multivariate Cox regression analysis was performed to address the influence of independent factors on disease-free survival (RFS) and overall survival (OS). In addition, 31 SKCM and 31 nevus tissues were collected for immunohistochemical (IHC) staining and evaluation. STRING, DAVID and Gene Set Enrichment Analysis (GSEA) was utilized to conduct a network of related genes and significant pathways. Furthermore, we investigated the relationship of A3G with tumor-infiltrating immune cells (TIICs) by TIMER and TISIDB.

**Result:** We found both transcriptional and proteomics expressions of A3G were elevated in SKCM. Survival analysis and ROC curves showed significant diagnostic and prognostic ability of A3G. IHC results showed increased expression of A3G in SKCM compared to nevus tissues. Importantly, A3G expression was closely associated with the immune-infiltrating levels of B cells, CD4+ T, CD8+ T, neutrophils, macrophages and dendritic cells.

**Conclusion:** In summary, our study first reveals that elevated A3G expression is significantly correlated with better prognosis in SKCM patients. The role of A3G in SKCM demonstrated that it might be a prognostic and immunotherapeutic biomarker for SKCM.

## Introduction

Skin cutaneous melanoma (SKCM) accounts for over 75% of skin cancer-related deaths each year 1, which becomes one of the most life-threatening malignancies damaging human health. The pathogenesis of SKCM, according to the Clark model, gives the assumption that the progression from melanocytes to malignant melanoma needs several steps, including formation of banal nevi, dysplastic nevi, melanoma *in situ*, and invasive melanoma 2. Nowadays, surgical resection is usually preferred to treat primary melanoma patients; however, advanced melanomas are much more aggressive and not sensitive to radiotherapy and chemotherapy 3. Increasing evidence showed that immunotherapies and targeted therapies, such as anti-PD-1, anti-PDL-1, anti-CTLA4 and MAGE-A3, can benefit the prognosis of patients with metastatic melanoma 4; while only a small part of patients can benefit from it 5. Thus, to better diagnose and treat melanoma patients, highly effective biomarkers are urgently needed.

The APOBEC (apolipoprotein B mRNA editing enzyme, catalytic polypeptide-like)/ AID (activation-induced cytidine deaminase) family is a group of cytidine deaminases that can convert cytosine (C) to uracil (U) in DNA/RNA 6. There are 11 members in the APOBEC family, including AID and APOBEC1, 2, 3(A/B/C/D/F/G/H), and 4 6. The APOBEC3 (A3) subfamily has been found to greatly participate in protecting cells from endogenous and exogenous DNA-based pathogens. Among them, APOBEC3G (A3G) was observed to inhibit the reverse HIV-1 and HBV transcription in infected cells, introducing C-to-T hypermutation in viral DNA 7, 8. Furthermore, the dysregulation of A3G is also involved in multiple tumorigeneses. For example, Takashi Iizuka et al. found that A3G is closely correlated with uterine cervical intraepithelial neoplasia (CIN) and might become an effective biomarker to assess CIN progression 9. Also, A3G was observed to drive tumorigenesis in hepatocellular carcinoma (HCC) and might mediate host innate resistance to HBV infection and HCC 10. Importantly, A3G is broadly expressed in human tissues, and mRNA levels of A3G widely associate with lymphoid cell content 11. Those findings all show that A3G plays a key role in human malignancies and immune infiltrates. While the relationship between A3G and melanoma is rarely studied currently.

Therefore, we explored the gene expression profiles, the potential prognostic value, and the underlying biological interaction networks of A3G in SKCM patients. Furthermore, we investigated the relationship of A3G with tumor-infiltrating immune cells (TIICs) and revealed their replaceable role of A3G in tumor-immune interactions in SKCM.

## Methods

### Patients and variables

Melanoma tissues (n=31) and nevi tissues (n=31) were collected in the First Affiliated Hospital of Soochow University (Suzhou, China) from March 2016 to August 2019. Tissue samples were pathologically confirmed and fixed in 4% paraformaldehyde, available from the tissue bank. This study got approval by the Independent Ethics Committee (IEC) of the First Affiliated Hospital of Soochow University and it was conducted after informed consent of each subject.

### Transcriptional expression of A3G

Tumor Immune Estimation Resource (TIMER) database 12 is a useful website to analyze immune infiltrations among different types of cancers. The differential expression of A3G between tumors and normal tissues could be explored cross all the TCGA (The Cancer Genome Atlas) database tumors. The distributions of A3G expression levels were shown with box plots, and the statistical significance was evaluated via the Wilcoxon test.

Gene Expression Profiling Interactive Analysis (GEPIA, http://gepia.cancer-pku.cn/) is a friendly online tool for users to explore customizable functionalities based on data from TCGA (https://tcga-data.nci.nih.gov/tcga/) and the Genotype-Tissue Expression project (GTEx; https://www.gtexportal.org/home/index.html) 13. GEPIA was used to identify the transcriptional expression of A3G between SKCM and normal tissues.

### The Human Protein Atlas

The Human Pathology Atlas project (https://www.proteinatlas.org) contains immunohistochemistry (IHC) data by using a tissue microarray-based analysis, including proteome analysis of 17 cancer types and 44 different normal tissue types 14. Staining intensity, location, quantity, as well as patients' information in different cancer types; were all available online. In the current study, we used the Human Pathology Atlas to investigate the protein expressions of A3G in SKCM and normal skin tissues.

### Survival Analysis

Survival analysis was performed based on gene expression levels by GEPIA, using the log-rank test for the hypothesis evaluation. Thus, disease free survival (RFS), as well as overall survival (OS) analysis, was constructed. The x-axis showed time in days, and the y-axis represented the proportion of people surviving. The lines showed survival curves of two groups, with high expression marked in red and low expression marked in blue. The dotted lines presented the 95% confidence interval information in the survival plots.

### Statistical analysis

A total of 481 SKCM patients with clinical profiles, among which 475 SKCM patients with available RNA-sequence data from the TCGA database, were collected in analyses. Cox regression models were used for univariate and multivariate analysis to identify independent variables, including age at diagnosis, gender (ref. Male), Breslow depth, Clark level (ref. I- III), pT stage (ref. T1- T2), pN stage (ref. N0), pM stage (ref. M0), pathological stage (ref. I-II), and APOBEC3G expression (ref. Low). P-values < 0.05 were considered significant in all tests. The integrated score was identified as sum of the weight of A3G and significant clinicopathological prognostic indicators.

### Immunohistochemistry (IHC)

Protein expression levels of A3G were measured using IHC staining and mouse monoclonal Anti-A3G antibody (ab75560). Positive or negative staining of a certain protein in one FFPE slide was independently assessed by two experienced pathologists and supervised by a clinician. Based on the level of staining intensity (no staining, weak, moderate and strong staining), the score ranged from 0 to 3 15. The staining extent was graded from 0 to 4 for the coverage percentage of immunoreactive tumor cells (0%, 1-25%, 26-50%, 51-75%, 76-100%). According to the multiply of the staining intensity and extent score, the total IHC score grading from 0 to 12 was evaluated. Negative staining represented grade 0 to 4 and positive staining from 5 to 12 for each sample.

### Genomic alteration of A3G

The cBioPortal (http://cbioportal.org) is a straightforward website that provides multidimensional cancer genomics datasets including more than 5000 tumor samples from 20 cancer studies 16. To study the A3G mutation in SKCM, the cBioPortal database was used. Genomic alteration types, alteration frequency as well as protein change in amino acid in SKCM were analyzed. The genomic alterations of A3G included copy number amplification, mRNA upregulation, deep deletion, missense mutation with unknown significance, and so on.

### Protein-protein interaction (PPI) network construction

In this study, STRING (http://string-db.org) (version 11.0) was adopted to explore the protein co-regulation of A3G and measure functional interactions between proteins 17. The interaction specificity score > 0.4 was considered statistically significant.

### Functional annotations

DAVID (http://david.ncifcrf.gov) (version 6.8) 18, 19 was applied to perform functional enrichment analysis, including the biological process (BP), cellular component (CC), molecular function (MF), visualized in the bubble chart. P-value<0.05 was considered statistically significant.

Next, we performed gene set enrichment analysis (GSEA) to predict potential hallmarks by using transcriptional sequences from TCGA database. A permutation test with 1000 times was applied to find the most significantly involved pathways 20. Adj. p < 0.01 and FDR < 0.25 were considered as significant related genes. Statistical analyses and graphical plotting were conducted using R software (version 3.3.2).

### Immune infiltration analysis

TIMER was then utilized to analyze the comprehensive correlation between A3G genes and tumor-infiltrating immune cells signatures. Furthermore, an integrated repository portal for tumor-immune system interactions (TISIDB, http://cis.hku.hk/TISIDB/index.php) 21 was adopted to show the tumor and immune system interactions among 28 types of tumor-infiltrating lymphocytes (TILs) in different types of tumors. Based on A3G expression profile, the relative abundance of TILs was inferred by using gene set variation analysis. Spearman's test was used to measure correlations between A3G and TILs. P-values < 0.05 were considered significant in all tests.

## Results

### Clinical and pathologic characteristics baseline of SKCM patients from TCGA and discovery cohort

481 SKCM patients were enrolled from the TCGA cohort, and 31 from the discovery cohort. Clinicopathological parameters of all patients, including age at surgery, gender, Clark level, Breslow depth, TNM stage and pathologic stage was shown in **Table 1**.

### The differential expression of A3G in various tumors

**Figure 1:**
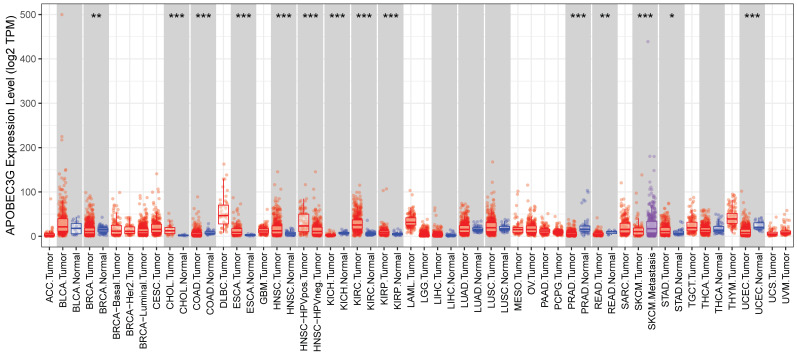
The expression of APOBEC3G in the cancerous tissues and in adjacent normal tissues. The APOBEC3G expression was analyzed in various cancerous tissues and adjacent normal tissues through the TIMER database. *p<0.05, **p<0.01, ***p < 0.001.

Due to the possibility that A3G might represent an important new biomarker for tumor diagnosis, we used the TIMER database to investigate the expression of A3G in different tumors and adjacent normal tissues in order to find out whether A3G expression associates with cancers. As shown in **Figure 1**, the expression of A3G was found to be higher in metastatic melanoma compared to primary melanoma. In addition, the elevated A3G expression was found in multiple tumor tissues compared with normal tissues, such as cholangiocarcinoma (CHOL), esophageal carcinoma (ESCA), head and neck squamous cell carcinoma (HNSC), kidney renal clear cell carcinoma (KIRC), kidney renal papillary cell carcinoma (KIRP), and stomach adenocarcinoma (STAD). While decreased expression of A3G was observed in several tumor tissues compared with adjacent normal tissues, including breast invasive carcinoma (BRCA), colon adenocarcinoma(COAD), kidney chromophore (KICH), prostate adenocarcinoma (PRAD), rectum adenocarcinoma (READ), and uterine corpus endometrial carcinoma (UCEC). The expression profiling of A3G indicated that it might be acting as different role in the development of various types of tumors.

### Differential expression of A3G in SKCM patients

**Figure 2:**
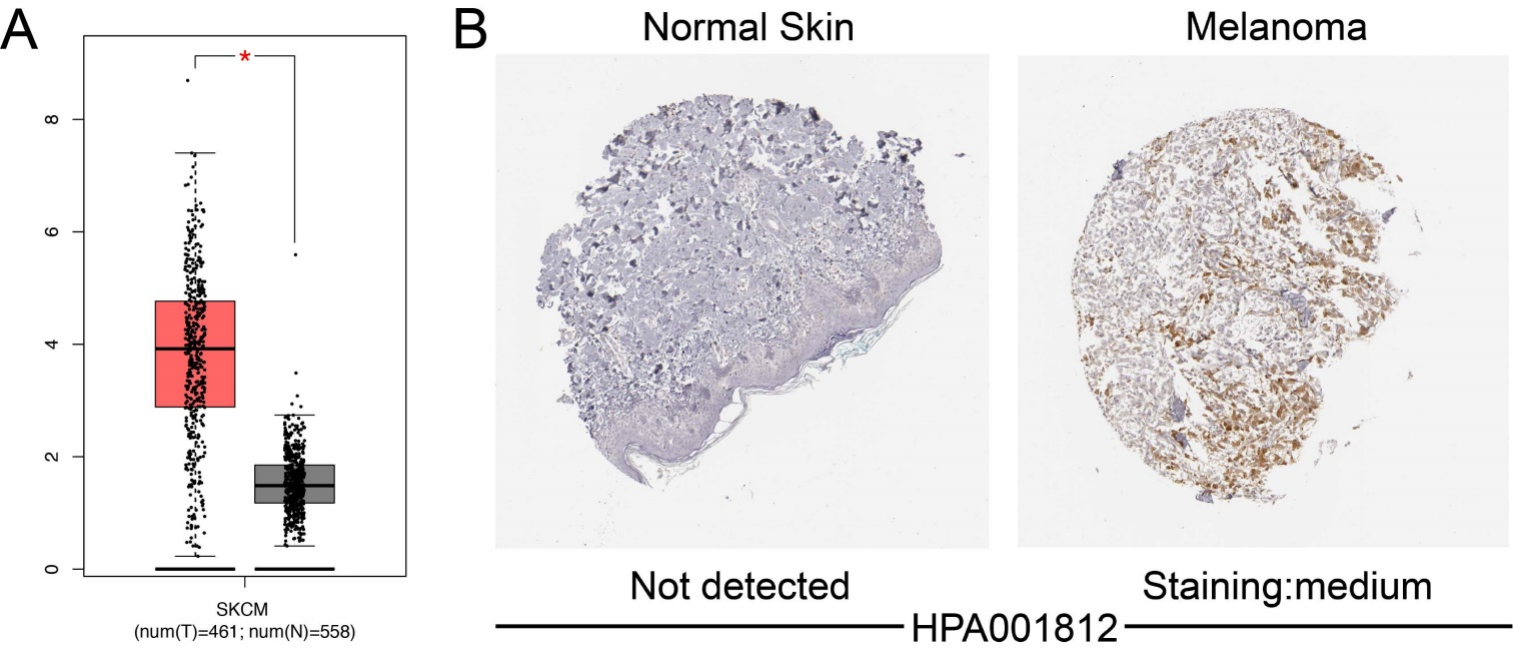
Differential APOBEC3G expression in SKCM tissues and adjacent normal tissues. **(A)** Transcriptional level of APOBEC3G expression was found highly expressed in SKCM tissues compared with normal tissues by GEPIA (*p < 0.05). **(B)** Medium expression of APOBEC3G is detected in melanoma tissues while no expression detected in normal tissues using the Human Protein Atlas.

Using GEPIA, we investigated the mRNA expression of A3G between SKCM samples and normal tissues based on data from TCGA and GTEx. mRNA level of A3G was highly expressed in SKCM tissues compared with normal tissues (p<0.05) (**Figure 2A).** IHC staining revealed that A3G staining was medium expressed in SKCM tissues, while expression was not detected in normal skin tissues (**Figure 2B**). Taken together, these findings indicated that A3G was highly expressed at both transcriptional and proteomic levels in SKCM tissues compared with normal tissues.

### Prognostic value of A3G in TCGA cohorts

**Figure 4:**
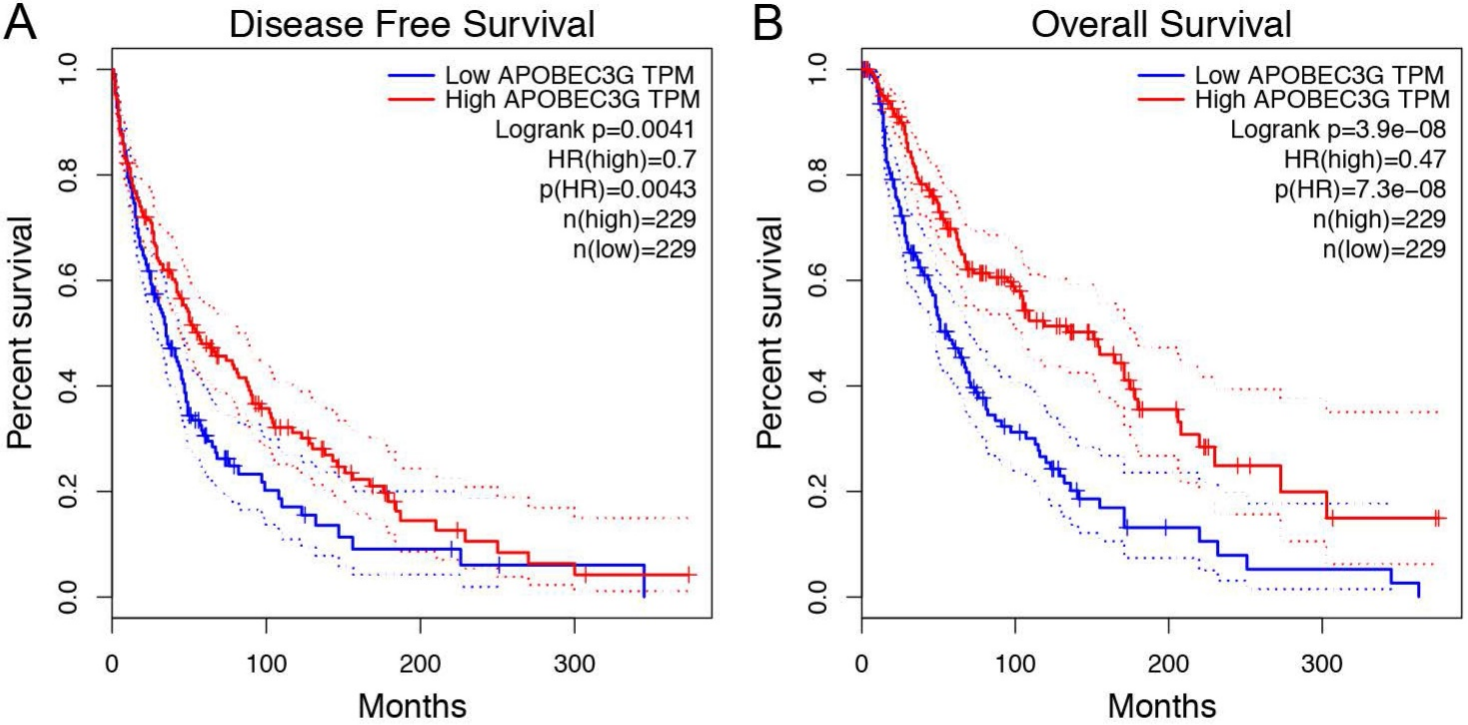
Survival analysis in Kaplan-Meier method indicated that APOBEC3G was significantly correlated with better RFS (p= 0.0041) and OS (p = 3.9e-08) in SKCM patients.

**Figure 5:**
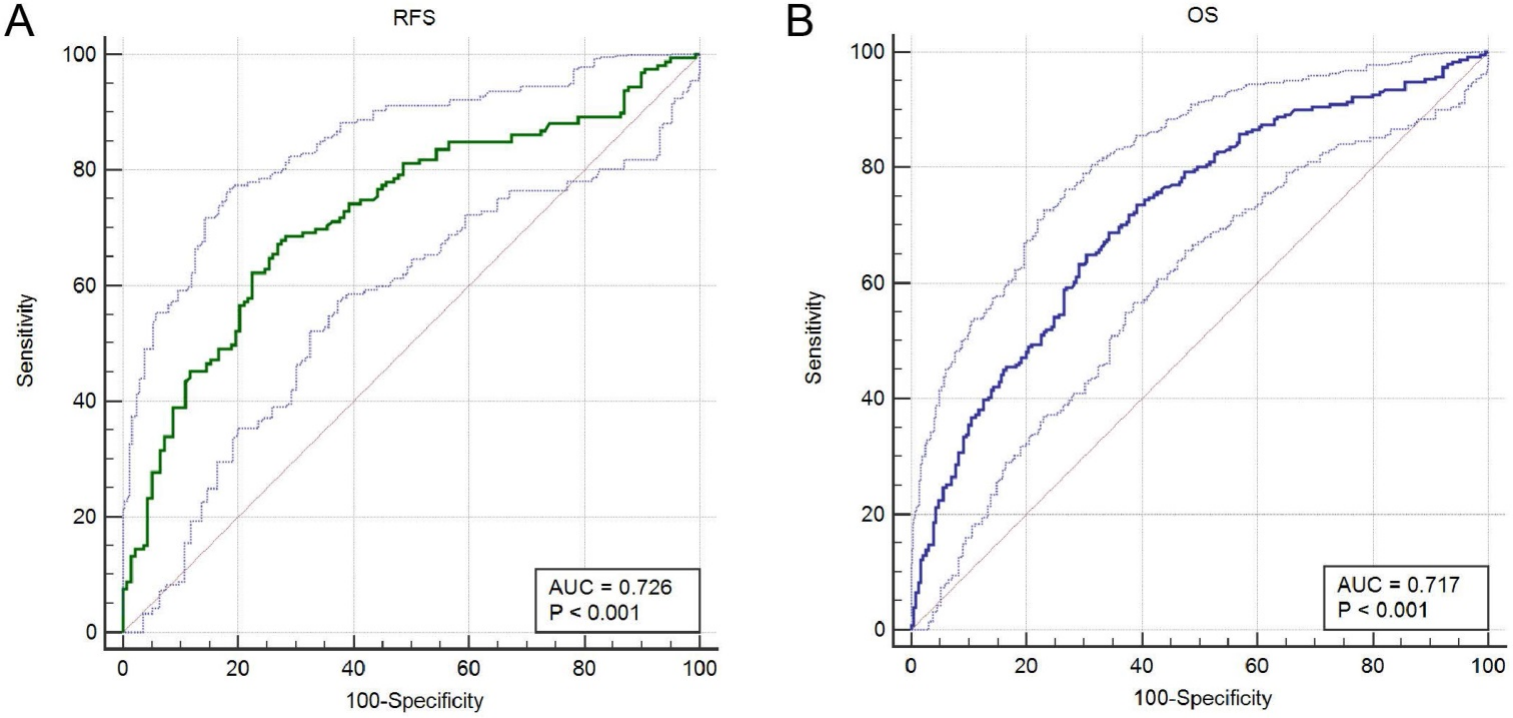
ROC curves were generated to validate the ability of the ROC model to predict prognosis. **(A)** The AUC index for the RFS were 0.726 (p < 0.001). **(B)** The AUC index for the OS were 0.717 (p < 0.001).

Prognostic analysis indicated that increased A3G expression was greatly associated with better RFS (p = 0.0041) and OS (p = 3.9e-08) in the TCGA cohort (**Figure 4A-B**). Furthermore, ROC curves were conducted to evaluate the ability of the gene model to predict prognosis events. After integrating all the significant clinicopathological parameters and gene expression profiles in the Cox regression models (**Table 2 and Table 3**), we generated the formula: 1.02×Age+2.26×pN stage (ref. N0)+2.28×pM stage (ref. M0)+ 0.59×APOBEC3G expression (ref. Low) for OS. The AUC indices for the RFS and OS were 0.726 and 0.717, respectively (p < 0.001;** Figure 5A-B**).

### IHC staining analyses in SKCM patients from discovery cohort

**Figure 6:**
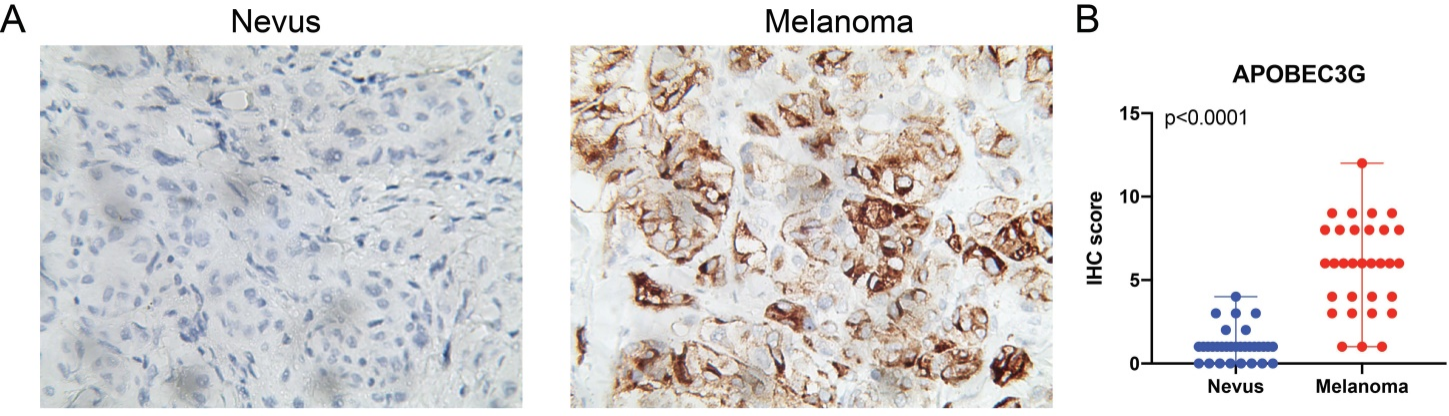
** (A)** IHC staining indicated significantly elevated APOBEC3G expression in terms of density and intensity in melanoma tissues compared with nevus tissues. **(B)** Scatter plots of IHC score between melanoma and nevus tissues were illustrated (P<0.0001).

Due to the fact that many of the melanomas are directly derived from nevus, we next validate differential expression of A3G between SKCM tissues and nevi tissues. IHC analysis was performed and we found significantly elevated A3G protein expression in the SKCM than in the nevi tissues. The results and the scatter plots of the IHC score (p<0.0001) were illustrated in **Figure 6.**

### Genomic alteration of A3G

**Figure 7:**
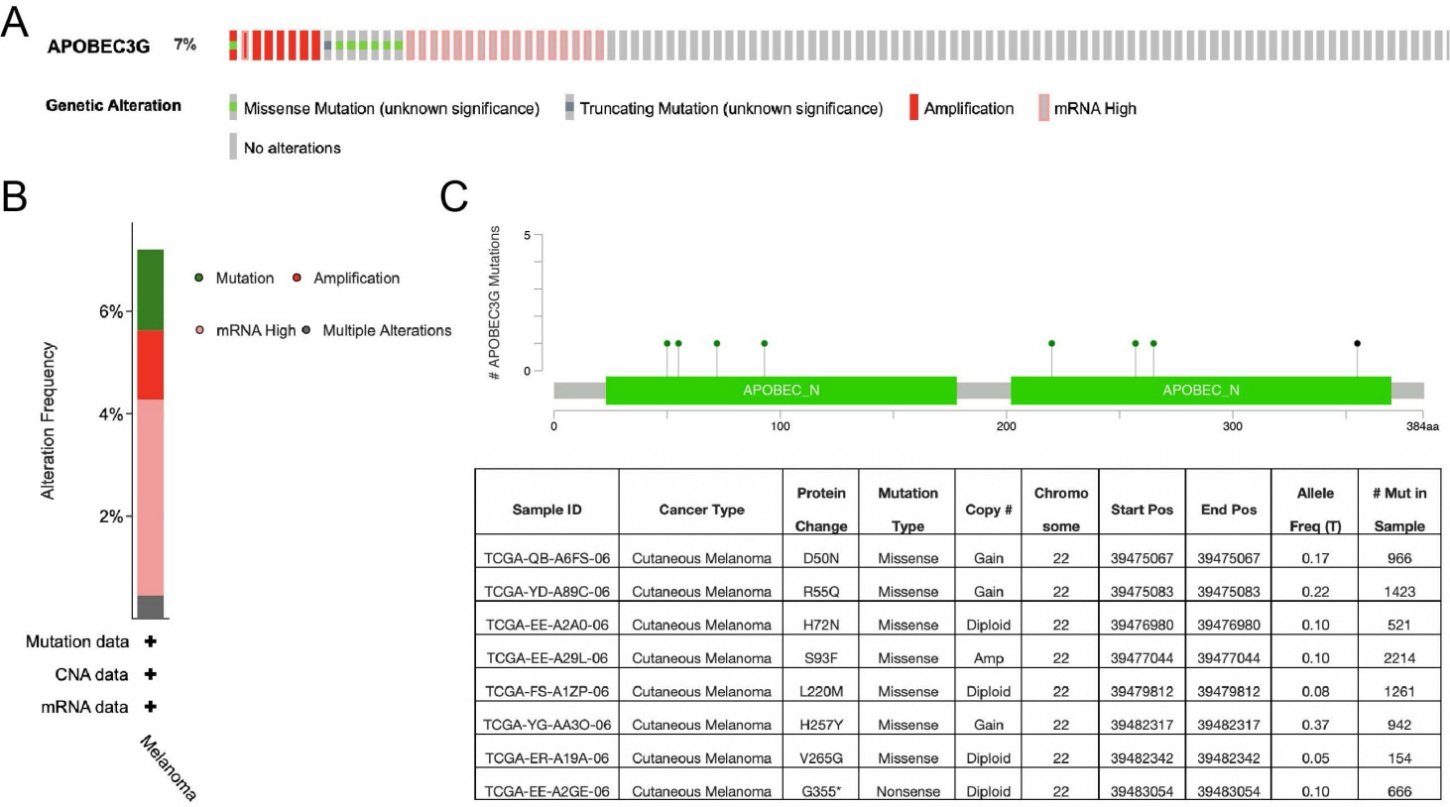
Genomic mutation of APOBEC3G in SKCM via cBioPortal database. **(A)** The genetic alterations of APOBEC3G in SKCM, including copy number amplification and deep deletion, missense mutation with unknown significance, and mRNA upregulation, and genomic mutation of APOBEC3G were assessed. **(B)** The genetic alteration type and frequency of APOBEC3G. **(C)** The mutation of APOBEC3G amino acids was analyzed.

We further explored the A3G alteration status by cBioPortal in SKCM. A total of 444 patient samples were included from TCGA database for analysis. The rate of alteration frequency in A3G was 7%. The most frequent alteration type was observed to be mRNA upregulation, followed by missense mutation, amplification and truncating mutation (**Figure 7A-B**). In addition, the details of A3G mutation that resulted in the amino acid change were illustrated in **Figure 7C**. The above results showed that genetic alteration of A3G could be found in SKCM, which might play an important role in the tumorigenesis of SKCM.

### Functional annotations of A3G

**Figure 8:**
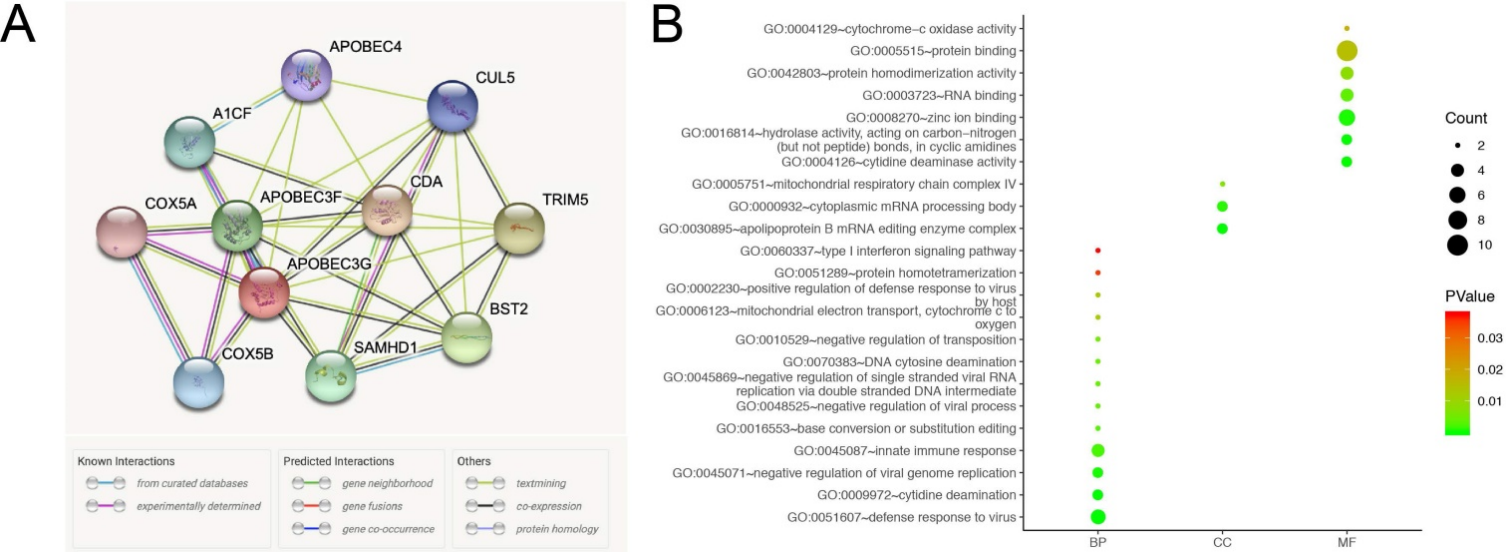
Functional annotations and predicted signaling pathways. **(A)** The PPI network of APOBEC3G was constructed. A network of APOBEC3G and its co‐expression genes was set up visually. **(B)** Functional enrichment analyses of a total of 11 involved genes were performed and visualized in bubble chart.

A network of A3G along with co-expression genes was illustrated in** Figure 8A.** As shown in **Figure 8B**, functional enrichment analyses were performed among involved genes, and then visualized in the bubble chart. GO analysis indicated that changes in biologic processes (BP) significantly enriched in defense response to virus, cytidine deamination, negative regulation of viral genome replication, innate immune response, base conversion or substitution editing, DNA cytosine deamination, type I interferon signaling pathway, and mitochondrial electron transport, cytochrome c to oxygen. Changes in cellular components (CC) were mainly enriched in the apolipoprotein B mRNA editing enzyme complex, cytoplasmic mRNA processing body, and mitochondrial respiratory chain complex IV. As for molecular function (MF), significant genes were primarily involved in protein binding, cytidine deaminase activity, hydrolase activity, acting on carbon-nitrogen (but not peptide) bonds, in cyclic amidines, RNA binding, protein homodimerization activity, and cytochrome-c oxidase activity.

### Significant genes and pathways obtained by GSEA

**Figure 9:**
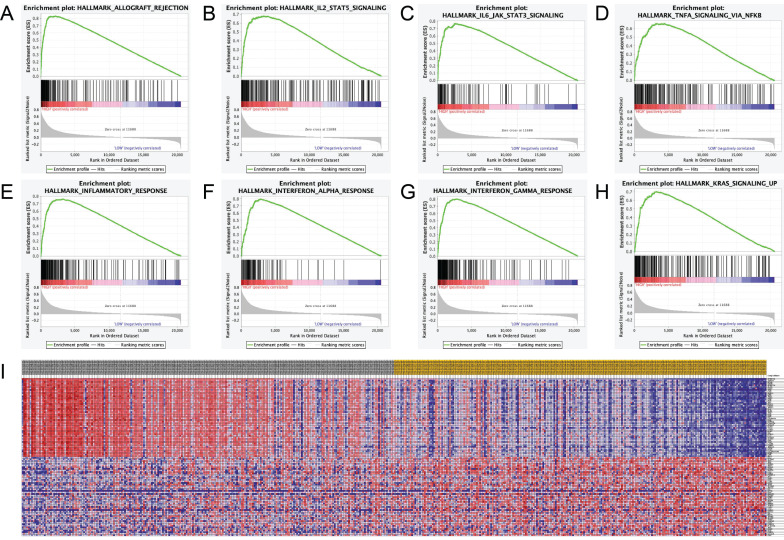
Significant related genes and hallmarks pathways in SKCM obtained by GSEA. **(A-H)** The most involved significant pathways included allograft rejection, IL2-STAT5 signaling, IL6/JAK-STAT3 signaling, TNF-A signaling via NF-κB, inflammatory response, interferon-alpha response, interferon-gamma response, and Kras signaling. **(I)** Transcriptional expression profiles of a total of 100 significant genes with positive and negative correlations were performed in a heat map.

Hallmark analysis for A3G was performed by using GSEA. The results revealed that the most involved significant pathways included allograft rejection, IL2-STAT5 signaling, IL6/JAK-STAT3 signaling, TNF-A signaling via NF-κB, inflammatory response, interferon-alpha response, interferon-gamma response, and Kras signaling. The details were shown in **Figure 9A-H**. Furthermore, the heat map showed transcriptional expression profiles of the 100 significant genes in **Figure 9I.**

### Correlation between A3G and immune infiltration level

**Figure 10:**
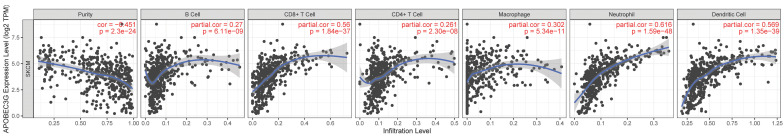
TIMER analysis demonstrated significant positive associations with infiltrating levels of B cell (r = 0.27, p = 6.11e-09), CD8^+^ T cells (r = 0.56, p = 1.84e-37), CD4^+^ T cells (r = 0.261, p = 2.30e-08), macrophages (r = 0.302, p = 5.34e-11), neutrophils (r = 0.616, p = 1.59e-48) and dendritic cell (r = 0.569, p = 1.35e-39) in SKCM.

**Figure 11:**
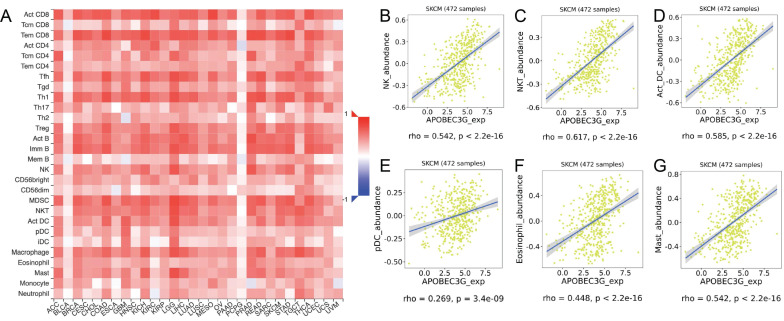
Correlations between expression of APOBEC3G and TILs. **(A)**Relations between expression of APOBEC3G and 28 types of TILs across human cancers. **(B-G)** APOBEC3G significantly correlated with abundance of natural killer cells (NK cells; rho = 0.542 p < 0.001), natural killer T cells (NK T cells; rho = 0.617, p < 0.001), activated dendritic cells (act DC, rho = 0.585, p < 0.001), plasmacytoid dendritic cells (pDC, rho = 0.585, p < 0.001), eosinophil (rho=0.448, p < 0.001) and mast cells (rho=0.542, p < 0.001).

TIMER was applied to comprehensively investigate the molecular feature of tumor-immune interactions in order to explore the effect of A3G expression in tumor microenvironment (TME) of melanoma. TIMER analysis revealed significant positive associations with infiltrating levels of B cell (r = 0.27, p = 6.11e-09), CD8^+^ T cells (r = 0.56, p = 1.84e-37), CD4^+^ T cells (r = 0.261, p = 2.30e-08), macrophages (r = 0.302, p = 5.34e-11), neutrophils (r = 0.616, p = 1.59e-48) and dendritic cell (r = 0.569, p = 1.35e-39) in SKCM (**Figure 10**). Additionally, we identified significant correlations of A3G with 28 types of TILs among human malignancies (**Figure 11A**). A3G greatly correlated with abundance of natural killer cells (NK cells; rho = 0.542 p < 0.001), natural killer T cells (NK T cells; rho = 0.617, p < 0.001), activated dendritic cells (act DC, rho = 0.585, p < 0.001), plasmacytoid dendritic cells (pDC, rho = 0.585, p < 0.001), eosinophil (rho=0.448, p < 0.001) and mast cells (rho=0.542, p < 0.001) in **Figure 11B-G**.

## Discussion

Mutation leads to genomic variation, thus possibly contribute to tumorigenesis, recurrence, and therapy resistance in human beings. It is proved that misregulation of A3s brings about somatic mutation in many types of cancers 6. Therefore, inhibiting these enzymes may be an effective way to prevent procancerous mutations from occurring, including those involved in tumor metastasis, recurrence, and drug resistance. Importantly, T cells are found to increase A3G expression, responding to viral infections like HIV. However, overexpression of A3G in infiltrated T cells was also found in non-virus related cancers. Brandon Leonard et al. reported that A3G expression levels in tumor infiltrating T lymphocytes may serve as a predictive biomarker for strong anti-cancer T cell responses and improved patients' outcomes in high-grade serous ovarian carcinoma 22. However, the relationship between A3G and melanoma remained unclear. In the present study, mRNA expression of A3G gene and survival of SKCM patients were obtained from multiple databases, analyzed to predict the function of A3G genes, and assessed for the potential role of the A3G mRNA expression to be utilized as useful prognosis biomarker. In addition, the IHC analyses between nevus and melanoma tissues were performed to further validate the bioinformatics results. Functional enrichment and GSEA analysis illustrated that A3G was significantly involved in the most significant hallmarks pathways including allograft rejection, IL2-STAT5 signaling, IL6/JAK-STAT3 signaling, TNF-A signaling via NF-κB, inflammatory response, interferon-alpha response, interferon-gamma response, and Kras signaling in SKCM samples.

Interferons (IFNs) play an important role in the generation of an anti-tumor immune response 23. IFN pathway participates in as well as predicts the response to inhibition of key oncogenic mutation MAPK in melanoma 24. IFN-α is extensively used as an adjuvant treatment in high-risk melanoma 25, 26. The mechanism behind IFN-α's role in melanoma is considered to be immunomodulatory rather than anti-angiogenic or cytotoxic 27. IFN-γ-mediated inflammation may contribute to an immunosuppressive and tolerogenic tissue or TME, which may be mechanistically achieved by IFN-γ-induced enhancement of immune checkpoints, mediated by molecules like PD-1 and PD- L1 in the TME 28. Furthermore, IFN-γ is assumed to be intimately associated with the elimination stage of the immunoediting paradigm 29. It was reported that IFNs can induce A3G expression on the uterine cervical squamous cells 9. In this study, A3G was found to participate in the interferon-alpha response and interferon-gamma response in SKCM patients, which might provide novel clues for SKCM treatments.

Inflammation can be found in common physiological processes and cancer related inflammation (CRI) is recognized as one of the hallmarks in the pathogenesis of many types of malignancies 30, 31. CRI is coordinated by inflammatory cells and mediators such as chemokines and cytokines on malignant and non-malignant cells, acting in an autocrine and paracrine manner 32. In addition, it predicts response to immune checkpoint blockade in human melanoma 33. Together with genetic alterations, the inflammatory tumor environment leads to progression and metastasis of tumors eventually 34. A3G in SKCM regulates a range of inflammation-related signaling pathways such as TNF-α signaling via the NF-κB pathway, IL2-STAT5 signaling, IL6/JAK-STAT3 signaling.

The role of TNF family is recognized as double-edged swords that regulate immune responses, haematopoiesis and morphogenesis, as well as tumorigenesis, viral replication, and rheumatoid arthritis 35. Previous research showed that TNF-α as one of the pro-inflammatory cytokines can function as potent inducers of melanoma cell plasticity in the context of T cell immunotherapy 36. Non-apoptotic TNF-family receptor Fas signaling is found to induce growth and migration of tumor cells, and weaken the efficacy of T cell adoptive immunotherapy 37. Furthermore, NF-κB transcription factors are vital to immune system activation and immune response upregulation to defend both foreign pathogens and cancers which can be activated by Fas under certain conditions 38, 39. In the present study, A3G was observed that participated in TNF-α signaling via NF-κB pathway from GSEA analysis, which might provide a novel strategy to improve anti-tumor immunity by blocking the non-apoptotic function of these receptors.

IL-2 was used as the first reproducible effective human cancer immunotherapies due to its ability to expand T cells with maintenance of functional activity 40. Currently, the use of cytokines from the IL-2 family (also known as the common gamma chain cytokine family) including IL-2, IL-15 and IL-21 to activate the immune systems of tumor patients becomes one of the most important fields in tumor immunotherapy research 41. IL-2 can stimulate cytotoxic T lymphocytes and is a longstanding treatment option for metastatic melanoma patients 42. Infusion of IL-2 in multiple cycles at different doses in metastatic melanoma patients proved that the immune system can completely eradicate cancer cells under certain conditions which have led to the first success in cancer immunotherapy 41. In our study, GSEA analysis demonstrated that A3G regulated the IL2-STAT5 signaling pathway in SKCM patients. The IL2-STAT5 signaling pathway can participate in immune-related anti-tumor effects, promotes proliferation of tumor cells, and interacts with other significant tumor-related pathways. Clinical application of IL-2 to exert anti-tumor effects while inhibiting the STAT5 signaling pathway might be an effective immunotherapy for SKCM.

Previous studies showed that the IL-6/JAK-STAT3 pathway is abnormally hyperactivated in multiple tumor types and elevated expression of IL‐6 can stimulate hyperactivation of JAK/STAT3 signaling which usually leads to an unfavorable clinical outcome 43-45. IL-6 activates STAT3 phosphorylation, inducing the transcription of genes that regulate tumor cell proliferation and antiapoptosis 46. Kushiro et al. reported that IL-6 can drive melanoma metastasis via its ability to promote melanoma cell invasion 47. In addition, upon tumor antigen recognition by T cells, JAK-STAT-mediated expression of PD-1 ligands PD-L1 and PD-L2 can be triggered by released interferons on the surface of melanoma cells 1, 45.

This study is the first one to identify the relationship between A3G expression and tumorigenesis or prognosis of SKCM. Notably, A3G promotes a series of immune responses and tumor environment, which are estimated to be highly expressed in many cancers. Therefore, multiple databases were used to explore the differential A3G expression between tumor and normal tissues, and the prognostic value of A3G in SKCM. Furthermore, we also constructed a PPI network of co-regulatory proteins. Functional enrichment analysis was performed among co-expression genes. GSEA analysis was implemented to investigate the most involved genes and hallmark pathways, which may shed light on the association that triggers carcinogenesis. In addition, merely expression level of A3G was identified in this study, thus further functional works, as well as validated cohorts, were needed to verify the absoluteness of these findings.

## Conclusion

In summary, this study first showed that increased A3G expression is significantly associated with better prognosis in SKCM patients. The most significant hallmark pathways of A3G involved IL2-STAT5 signaling, IL6/JAK-STAT3 signaling, inflammatory response, interferon-alpha response, interferon-gamma response, Kras signaling and TNF- alpha signaling via NF-κB. Furthermore, the expression of A3G was also closely correlated with immune infiltration. Thus, our findings present a significant role of A3G in tumor-immune interactions and might serve as a promising prognostic and immunotherapeutic biomarker in SKCM. However, further studies are required to elucidate molecular pathogenesis and alteration in signaling pathways of A3G in melanoma.

## Figures and Tables

**Figure 3:**
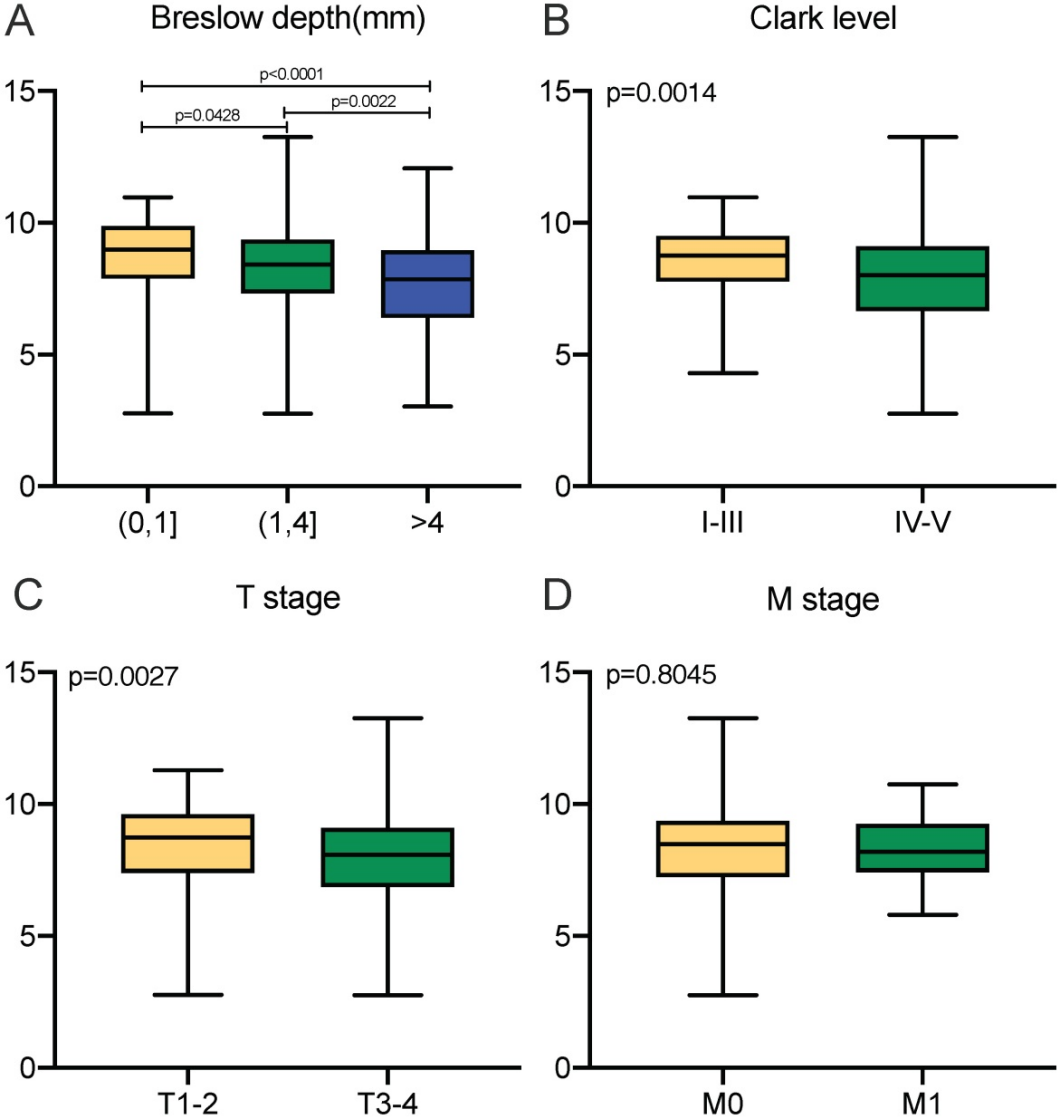
Transcriptional expressions of APOBEC3G significantly correlated with clinicopathological parameters in SKCM patients from TCGA cohort. **(A-C)** Transcriptional expression of APOBEC3G was significantly correlated with Breslow depth, Clark level and T stage (p<0.05). **(D)** Transcriptional expression of APOBEC3G was not greatly correlated with M stage (p=0.8045).

**Table 1:** Clinicopathological characteristics of SKCM patients

Characteristics	Discovery cohort (N=31)	TCGA cohort (N=481)
N (%)		
**Age**		
≤60 years	13 (41.9)	258 (54.7)
>60 years	18 (58.1)	214 (45.3)
**Gender**		
Male	20 (64.5)	297 (61.9)
Female	11 (35.5)	183 (38.1)
**Clark level**		
I	13 (41.9)	6 (1.8)
II	15 (48.4)	18 (5.5)
III - IV	3 (9.7)	246 (75.5)
V	0 (0)	56 (17.2)
**Breslow depth (mm)**		
≤0.75	5 (16.1)	36 (10.2)
0.76-1.50	10 (32.2)	65 (18.4)
1.51-4.00	13 (41.9)	106 (30.0)
>4.00	3 (9.8)	146 (41.4)
**pT stage**		
T1-T2	19 (61.3)	121 (32.7)
T3-T4	12 (38.7)	249 (67.3)
**pN stage**		
N0	31 (100)	236 (65.0)
N1	0 (0)	75 (20.7)
N2	0 (0)	52 (14.3)
**pM stage**		
M0	31 (100)	424 (94.4)
M1	0 (0)	25 (5.6)
**Pathologic stage**		
I- II	31 (100)	233 (53.9)
III-IV	0 (0)	199 (46.1)
**Persistent distant metastasis**		
No	31 (100)	217 (46.1)
Yes	0 (0)	254 (53.9)

**Table 2:** Univariate and multivariate Cox regression analysis of RFS in TCGA cohort

Covariates	Univariate analysis			Multivariate analysis		
	HR	95%CI	P value	HR	95%CI	P value
Age	1.02	1.003-1.031	0.02	1.01	0.989-1.026	0.43
Gender (ref. Male)	0.66	0.413-1.064	0.09	-	-	-
breslow depth	1.03	1.014-1.050	0.00	1.02	0.994-1.051	0.13
Clark level (ref. I-III)	1.87	1.054-3.303	0.03	0.95	0.5-1.802	0.88
pT stage (ref. T1-T2)	2.26	1.458-3.488	0.00	1.45	0.789-2.648	0.23
pN stage (ref. N0)	1.65	1.072-2.541	0.02	2.22	0.803-6.164	0.12
pM stage (ref. M0)	2.64	1.317-5.311	0.01	3.49	1.255-9.684	0.02
pathological stage (ref. I-II)	1.68	1.097-2.586	0.02	1.14	0.411-3.150	0.81
APOBEC3G expression (ref. low)	2.21	1.440-3.382	0.00	0.67	0.38-1.171	0.16

**Table 3:** Univariate and multivariate Cox regression analysis of OS in TCGA cohort

Covariates	Univariate analysis			Multivariate analysis		
	HR	95%CI	P value	HR	95%CI	P value
Age	1.02	1.015-1.034	0.00	1.02	1.005-1.028	0.00
Gender (ref. Male)	0.87	0.654-1.148	0.32	-	-	-
breslow depth	1.03	1.015-1.041	0.00	1.02	0.999-1.033	0.06
Clark level (ref. I-III)	2.13	1.499-3.015	0.00	1.26	0.836-1.898	0.27
pT stage (ref. T1-T2)	1.99	1.508-2.619	0.00	1.34	0.926-1.942	0.12
pN stage (ref. N0)	1.67	1.267-2.197	0.00	2.26	1.234-4.128	0.01
pM stage (ref. M0)	1.89	1.052-3.399	0.03	2.28	1.041-4.977	0.04
pathological stage (ref. I-II)	1.66	1.259-2.182	0.00	0.89	0.486-1.635	0.71
APOBEC3G expression (ref. low)	2.23	1.703-2.930	0.00	0.59	0.428-0.823	0.00
